# Global health inequities: a call for papers

**DOI:** 10.2471/BLT.23.289906

**Published:** 2023-04-01

**Authors:** Viroj Tangcharoensathien, Nisachol Cetthakrikul, Angkana Lekagu, Sasivimol Ontong, Rapeepong Suphanchaimat, Walaiporn Patcharanarumol

**Affiliations:** a International Health Policy Program, Ministry of Public Health, Tivanond Road, Nonthaburi, Thailand 11000.

Health inequities are systematic differences in the opportunity different groups of people have to achieve optimal health, leading to unfair and avoidable differences in health outcomes.^1^ Equity is critical for achieving the sustainable development goals (SDGs); furthermore, improving access to health care, education and basic services can reduce inequalities and promote economic growth. The United Nations’ *Sustainable development goals report 2022*^2^ reveals that the coronavirus disease 2019 (COVID-19) pandemic seems to have reversed the positive trend of reducing income inequality among countries – one of the targets of SDG 10.^2^ The report also shows that women and persons with disabilities are at heightened risk of discrimination.^2^ Inequalities between countries as well as conflicts and climate change affect migration of people. In 2021, the number of refugees worldwide reached the highest absolute number on record, and 5895 migrants died during the journey from their home countries to seek refuge or better life prospects in other countries.^2^ Migration of health workers from countries with a workforce deficit is a significant challenge that disrupts these countries’ provision of health services. To address this challenge, the World Health Organization (WHO) has developed the Health Workforce Support and Safeguards List 2020, which identifies 47 countries that should be provided with safeguards from active international recruitment of health personnel.^3^

Target SDG 10.6, “ensuring enhanced representation and voice for developing countries in decision-making in global international economic and financial institutions in order to deliver more effective, credible, accountable and legitimate institutions,”^4^ could also be applied to health institutions. How do different health institutions within a country and at an international level collaborate? How does the complex and crowded global health architecture in general look and work, beyond the current efforts in addressing health emergencies? These questions warrant a thorough investigation.

Health inequities can be affected by geopolitical determinants, that is, those related to governments, geographies, policies and the interests of countries and the relationship between them.^5^ For example, these determinants can affect the global health agenda-setting such as trade agreements influencing health outcomes of involved populations. Using trade agreements, industries that produce harmful products have tried to counteract WHO’s recommendations on, for example, formula milk marketing and tobacco and alcohol consumption, decreasing countries’ abilities to regulate in the interest of public health.^6^^–^^8^ Trade policies, political conflicts and resource allocation decisions can create barriers to access to essential medicines and health services, and exacerbate health disparities, particularly for marginalized populations. Conversely, equitable and sustainable health-care systems can contribute to peace, stability and economic development, leading to improved health outcomes for all. 

The *Bulletin of the World Health Organization* calls for papers that address inequities in global health. The first area is lessons learnt from various multilateral collaborations and their impacts on peace, stability, health systems development and the health of populations. The second area is the links between geopolitical factors and global health governance, agenda- setting, trust, voice and relationships between low- and middle-income countries, and high-income countries in global health decision-making.

The *Bulletin* welcomes contributions from stakeholders, public health decision-makers, researchers, and civil society and community representatives. Articles that address the impacts and solutions of inequities between countries and geopolitical determinants of health are welcome. We encourage manuscripts that identify effective and feasible policies that support effective political collaboration.

The deadline for submissions is 1 July 2023. Manuscripts should be submitted in accordance with the *Bulletin’s *guidelines for contributors (available at: https://www.who.int/publications/journals/bulletin/contributors/guidelines-for-contributors) and the cover letter should mention this call for papers. This theme issue will be launched at the Prince Mahidol Award Conference in February 2024.

## References

[1] Chapter 3. The root causes of health inequity. In: National Academies of Sciences, Engineering, and Medicine; Health and Medicine Division; Board on Population Health and Public Health Practice; Committee on Community-Based Solutions to Promote Health Equity in the United States; Baciu A, Negussie Y, Geller A, et al., editors. Communities in action: pathways to health equity. Washington, DC: National Academies Press; 2017. Available from: https://www.ncbi.nlm.nih.gov/books/NBK425845/ [cited 2023 Mar 5]. 28418632

[2] The sustainable development goals report 2022. New York: United Nations; 2022. Available from: https://unstats.un.org/sdgs/report/2022/The-Sustainable-Development-Goals-Report-2022.pdf [cited 2023 Mar 5]

[3] Health workforce support and safeguards list. Geneva: World Health Organization; 2020. Available from: https://cdn.who.int/media/docs/default-source/health-workforce/hwf-support-and-safeguards-list8jan.pdf?sfvrsn=1a16bc6f_14 [cited 2023 March 2]. 10.2471/BLT.23.290191 PMC10225944 37265683

[4] Goal 10: Reduce inequality within and among countries. New York: United Nations; 2015. Available from: https://www.un.org/sustainabledevelopment/inequality/ [cited 2023 Mar 6].

[5] Persaud A, Day G, Gupta S, Ventriglio A, Ruiz R, Chumakov E, et al. Geopolitical factors and mental health I. Int J Soc Psychiatry. 2018 Dec;64(8):778–85. 10.1177/0020764018808548 30760092

[6] Barlow P, Gleeson D, O’Brien P, Labonte R. Industry influence over global alcohol policies via the World Trade Organization: a qualitative analysis of discussions on alcohol health warning labelling, 2010-19. Lancet Glob Health. 2022 Mar;10(3):e429–37. 10.1016/S2214-109X(21)00570-2 35120586

[7] Mohamed SF, Juma P, Asiki G, Kyobutungi C. Facilitators and barriers in the formulation and implementation of tobacco control policies in Kenya: a qualitative study. BMC Public Health. 2018 Aug 15;18(S1) Suppl 1:960. 10.1186/s12889-018-5830-x 30168390 PMC6117633

[8] Russ K, Baker P, Byrd M, Kang M, Siregar RN, Zahid H, et al. What you don’t know about the codex can hurt you: how trade policy trumps global health governance in infant and young child nutrition. Int J Health Policy Manag. 2021 Dec 1;10(12):983–97. 10.34172/ijhpm.2021.109 34634868 PMC9309967

